# Awareness shaping or shaped by prediction and postdiction: Editorial

**DOI:** 10.3389/fpsyg.2015.00166

**Published:** 2015-02-18

**Authors:** Yuki Yamada, Takahiro Kawabe, Makoto Miyazaki

**Affiliations:** ^1^Faculty of Arts and Science, Kyushu UniversityFukuoka, Japan; ^2^Human Information Science Laboratory, NTT Communication Science Laboratories, Nippon Telegraph and Telephone CorporationAtsugi, Japan; ^3^Research Institute for Time Studies, Yamaguchi UniversityYamaguchi, Japan

**Keywords:** consciousness, vision, audition, touch, motor control, action, motion perception

Our conscious experience of the external world and/or our body states is quite rich. For example, we see the red color of a ripe apple, hear the sound of a stream, and feel the smoothness of silk by touch. In addition to the external world, we consciously experience the movement and states of our body. We intuitively believe that we are aware of all the events that occur in the external world, and that we control our body movements at will. From a scientific point of view, however, this is not true. Because of capacity limitations in neural processing, the brain can handle only a limited amount of information at once, and hence we experience just a fraction of available sensory inputs (e.g., change blindness: Rensink et al., 1997). The selected information does not necessarily shape our conscious experience as-is. To generate coherent perceptual representations of the external world/our body, the spatiotemporal integration and organization of the selected information is necessary.

However, neural processing in the brain inevitably takes a certain amount of physical time. Thus, this neural processing time should cause delays in our conscious experience from the actual transition of the external world/our body states. However, in general, we do not experience such temporal lags. One possibility is that the brain compensates for the lag and keeps up with the transition. How does the brain accomplish this seemingly difficult task?

Here we focus on the two strategies that the brain seems to adopt: “prediction,” which is the expectancy of an event that will arise in the future, and “postdiction,” which is a process that retrospectively interprets an event based on information available after the event (e.g., backward referral in Libet et al., 1979). How these two processes contribute to the generation of conscious experience has been an important question to date. Moreover, it is an intriguing question as to how these processes, prediction and postdiction, interact with each other in shaping conscious experience.

The present research topic aims at contributing to the understanding of the neural and psychological mechanisms underlying the generation of conscious experience. To this end, we collected the latest research focusing on the role of the temporal aspects of neural processing, such as prediction and postdiction, in shaping conscious experience. Additionally, we called the latest studies investigating the relation between conscious experience and spatial perception/sensorimotor factors. We present a brief overview of the research that this research topic includes.

First, the present research topic contains studies about the interaction between prediction and postdiction. Lenkic and Enns (2013) investigated the importance of both predictive and postdictive mechanisms in determining a target's shape visibility in an apparent motion sequence, and demonstrated that the postdictive influence was stronger than the predictive one. Hidaka and Nagai (2013) showed that a visual target in apparent motion was mislocalized by the offset signals of the target, and suggested that motion and position information are integrated in a postdictive manner. Vaughn and Eagleman (2013) showed that the Hering illusion was induced by radial optic flow in both predictive and postdictive (“peri-dictive”) manners, and discussed how the spatial warping counteracts processing lags. These studies psychologically suggest that conscious experience is generated by the temporal integration of sensory inputs. In addition, Goldreich and Tong (2013) provided a computational model that incorporates prediction and postdiction, which can broadly explain the cutaneous rabbit illusion and its related phenomena. The interaction between prediction and postdiction is not confined to the processing of a single modality, but rather extends to multiple modalities; e.g., Chien et al. (2013) showed that the perceived offset position of a moving object was modulated by temporally preceding/trailing sounds.

Integrating sensory signals across space as well as time is also an important component in generating our conscious experience. Roach and Webb (2013) showed that a tilt aftereffect induced by an implied orientation structure occurred even when the fringe of an occluded area was surrounded by a random orientation texture, suggesting integration of orientation gradients within extensive visual space.

This research topic includes reports that investigate the sensorimotor aspects of conscious experience. Synofzik et al. (2013) hypothesized that the sense of agency is established based on a complex interactive mechanism consisting of predictive and postdictive cues at sensorimotor, cognitive and affective levels. Sonoda et al. (2013) discussed the emergent nature of the sense of agency in terms of the observational heterarchical model. Ichikawa and Masakura (2013) showed that the flash-lag effect in the luminance dimension was modulated, depending on the sense of agency of manual control of the target's luminance change. It is intriguing to interpret this finding in the light of Synofzik et al.'s and Sonoda et al.'s models. Additionally, Higuchi (2013) reviewed behavioral studies regarding the anticipatory (i.e., predictive) nature of human locomotion. This review showed that visual information plays a critical role in modifying locomotor actions in an anticipatory manner in response to altered environmental properties. Honda et al. (2013) demonstrated that object-mass overestimation based on visual feedback delay (Di Luca et al., 2011) is determined by prediction errors in feedback timing rather than actual delays in visual feedback, suggesting that predictive mechanisms are involved in shaping awareness of object-masses.

Other theoretical considerations were also made. Bachmann (2012) provided a framework based on his perceptual retouch theory (e.g., Bachmann, 1984) in which interactions within and between stimulus-specific and non-specific processes in binding systems form conscious perception. In a review of Hubbard (2013), representational momentum was compared with the flash-lag effect in detail in terms of an extrapolation mechanism. Shimojo (2014) provided an extensive review on postdiction, encompassing sensorimotor, memory, and cognitive phenomena. The review has implications for underlying psychological and neural mechanisms and for explanations of real-world examples of postdiction.

As outlined above, a total of 14 articles written by 37 expert researchers across broad research areas discussed this topic from a variety of perspectives. We believe that these articles give researchers profound insights into how prediction and postdiction involve awareness of the external world and body states, and that the frameworks and findings provided here will serve to open up new avenues for future research.

## Conflict of interest statement

The authors declare that the research was conducted in the absence of any commercial or financial relationships that could be construed as a potential conflict of interest.
